# Improving Regulation of Enzymatic and Non-Enzymatic Antioxidants and Stress-Related Gene Stimulation in *Cucumber mosaic cucumovirus*-Infected Cucumber Plants Treated with Glycine Betaine, Chitosan and Combination

**DOI:** 10.3390/molecules25102341

**Published:** 2020-05-17

**Authors:** Ahmed R. Sofy, Rehab A. Dawoud, Mahmoud R. Sofy, Heba I. Mohamed, Ahmed A. Hmed, Noha K. El-Dougdoug

**Affiliations:** 1 Botany and Microbiology Department, Faculty of Science, Al-Azhar University, Nasr City, Cairo 11884, Egypt; ahmed_hmed@azhar.edu.eg; 2 Department of Virus Research, Plant Pathology Research Institute, ARC, Giza 12619, Egypt; rehab_dawood2011@yahoo.com; 3 Department of Biology, Faculty of Science, Jazan University, Box 114, Jazan 45142, Saudi Arabia; 4 Department of Biological and Geological Sciences, Faculty of Education, Cairo 11566, Egypt; 5 Botany and Microbiology Department, Faculty of Science, Benha University, Benha 13518, Egypt; nohaeldougdoug@gmail.com

**Keywords:** CMV, *Cucumis sativus*, antiviral, systemic acquired resistance, antioxidant defense systems, growth indices, osmoprotective

## Abstract

*Cucumber mosaic cucumovirus* (CMV) is a deadly plant virus that results in crop-yield losses with serious economic consequences. In recent years, environmentally friendly components have been developed to manage crop diseases as alternatives to chemical pesticides, including the use of natural compounds such as glycine betaine (GB) and chitosan (CHT), either alone or in combination. In the present study, the leaves of the cucumber plants were foliar-sprayed with GB and CHT—either alone or in combination—to evaluate their ability to induce resistance against CMV. The results showed a significant reduction in disease severity and CMV accumulation in plants treated with GB and CHT, either alone or in combination, compared to untreated plants (challenge control). In every treatment, growth indices, leaf chlorophylls content, phytohormones (i.e., indole acetic acid, gibberellic acid, salicylic acid and jasmonic acid), endogenous osmoprotectants (i.e., proline, soluble sugars and glycine betaine), non-enzymatic antioxidants (i.e., ascorbic acid, glutathione and phenols) and enzymatic antioxidants (i.e., superoxide dismutase, peroxidase, polyphenol oxidase, catalase, lipoxygenase, ascorbate peroxidase, glutathione reductase, chitinase and β-1,3 glucanase) of virus-infected plants were significantly increased. On the other hand, malondialdehyde and abscisic acid contents have been significantly reduced. Based on a gene expression study, all treated plants exhibited increased expression levels of some regulatory defense genes such as *PR1* and *PAL1*. In conclusion, the combination of GB and CHT is the most effective treatment in alleviated virus infection. To our knowledge, this is the first report to demonstrate the induction of systemic resistance against CMV by using GB.

## 1. Introduction

*Cucumber mosaic cucumovirus* (CMV) is one of the most common pathogens in the family Bromoviridae, belonging to the genus *Cucumovirus*. It is an economically crucial virus of agricultural crops. It has the largest range of host species known for any plant virus with approximately 1000 susceptible plant species [1]. It was present in all parts of the world, comprising various strains. Rapid adaptation was very successful for new hosts and new environments [2]. Depending on the strain of the virus and the host, CMV causes a broad range of symptoms, ranging from mild to extreme leaf mosaic or yellowing to stunting, chlorosis, necrosis and fruit distortion [3].

The physiological responses of stressed plants with virus infection are changed, with changes in energy metabolism, biomolecules including photosynthetic pigments, carbohydrates, lipids, proteins and signal transduction [4,5,6,7]. Virus-infected plants exhibit changes in physiological color, such as yellowing and chlorosis, suggesting that the virus affects the plant’s photosynthetic apparatus [5,8]. Increased generation of the reactive oxygen species (ROS) was also reported in virus-infected plants, causing oxidative stress [9,10,11]. Plant cells are defended against oxidative damage caused by ROS through the production of antioxidants such as ascorbic acid and antioxidant enzymes such as superoxide dismutase (SOD), catalases (CAT), peroxidase (POX) and phenylalanine ammonia lyase (PAL) [11].

More recent research has shown that the changes in the secondary metabolites such as phenols and flavonoids have played a key role in the host–pathogen interaction, disease development and infected plant defense reactions [12,13,14,15,16].

Therefore, the control of plant viral diseases is of great importance in both defense and treatment protocols. Application of pesticides for crop protection has been widely used since the last decades and has contributed to a substantial increase in farm yields, but pesticides can contaminate the aquifer’s environmental matrices, causing direct and permanent ecosystem damage [17,18,19,20]. Additionally, there is a real possibility that their residues could reach the consumers’ food chains [17]. The widespread use of such chemicals has also encouraged the development of resistance in significant crop pests [21]. No chemical antivirals have been able to completely cure virus-infected plants, so the researchers are trying to pursue alternative approaches by using substances that are environmentally sustainable, cost-effective and reproducible to help manage viral diseases such as glycine betaine (GB) and chitosan (CHT) [22].

GB (*N*,*N*,*N*-trimethyl glycine) is one of the most commonly osmoprotective compounds that contributes to the osmotic modification of all microorganisms and plants [22]. GB stabilizes the structure and function of the enzyme, as well as the integrity of the membrane against salt/osmotic stress [23]. In addition, GB has found to produce a defense mechanism by inducing antioxidant reactions under conditions of biotic stress [24]. However, there are no reports of the protective role of GB in the efficacy of biocontrol for CMV and other viral infections, but a few reports of its role in alleviating plant diseases and pathogens. Biocontrol activity against *Penicillium* *expansum* and *Botrytis cinerea* in *Candida oleophila* treated with GB was carried out [25]. Less aggregation of ROS and lower levels of cellular and lipid–protein oxidative damage was observed in *Candida oleophila* cells treated with GB [25]. In addition, Zhang, et al. [26] found that *Pichia caribbica* treated with GB showed an increase in biocontrol activity against blue mold on apples by decreasing the incidence of disease by 48.81% to 32.14% and oxidative stress tolerance, as well as improving the growth of apple wounds. GB treatment reduced accumulation of ROS, oxidative damage to cellular proteins and lipids in *P. caribbica* [26].

CHT has a wide variety of unique bioactivities, such as inducing plant resistance to viral infections, inhibiting viral infections in plant cells and preventing the development of phage infection in infected microbial cultures [27,28]. In both monocotyledons and dicotyledons plants, CHT induces host defense responses. These responses such as lignification of the cell wall [29], difference in the concentrations of ions, cytoplasm acidization, depolarization of membranes and phosphorylation of proteins [30], activation of chitinase and glucanase enzymes [31], phytoalexins biosynthesis [32], ROS production [33] and biosynthesis of phytohormones such as jasmonic acid and salicylic acid [34].

Cucumber (*Cucumis sativus* L.) is the main field and greenhouse vegetable crop in the Mediterranean Basin and the Middle East coastal areas [4]. Family *Cucurbitaceae* contains about 90 genera. Cucumber is one of the most popular cucurbitaceous crops in Egypt and is the leading vegetable export. Unfortunately, several pathogens may infect cucumber plants, and CMV is one of the main viruses that infect these plants in Egypt [4,35,36,37,38,39].

The objective of this investigation is, therefore, to study the efficacy of GB and CHT either alone or in combination in the protection of cucumber plants against CMV infection and their effects on plant growth, osmolytes, phenol content, the protective antioxidant and the expression of pathogenesis-related genes (*PR1* and *PAL1*).

## 2. Results

### 2.1. Disease Severity and Reduction of Virus Infectivity

**Figure 1:**
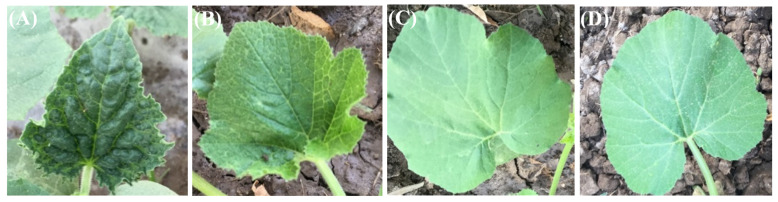
*Cucumber mosaic cucumovirus* (CMV) symptoms in untreated cucumber (challenge control) (**A**) and cucumber foliar-sprayed with glycine betaine (GB) (**B**), chitosan (CHT) (**C**) and GB+CHT (**D**) at 21 days after inoculation.

CMV-infected cucumber leaves (*Cucumis sativus* L. cv. Beta–alpha) showed severe symptoms compared to healthy leaves. Symptoms of CMV-infected leaves included severe mosaic, blisters and malformation (Figure 1A).

The application of exogenous GB and CHT either alone or in combination (GB+CHT) reduced the appearance of harmful symptoms caused by the development of the virus, especially when the leaves were treated with GB+CHT (Figure 1D). Mild symptoms of CMV have been seen by the spraying of leaves with GB, including mild mosaic and vein clearing (Figure 1B), and the foliar spraying of leaves with CHT has shown symptoms of vein clearing (Figure 1C), while the GB+CHT-treated leaves have not identified symptoms (Figure 1D).

CMV accumulation in systemically infected cucumber leaves was measured by ELISA (Table 1), where it decreased significantly in plants treated with GB, CHT and GB+CHT compared to challenge control (ChC). The most pronounced effect was achieved by applying the combination GB+CHT followed by CHT alone and GB alone.

The highest percentage of infection with CMV (95%) and the highest disease severity (70%) were recorded in challenge control plants (ChC) (Table 1). However, the use of GB and CHT showed that the infection decreased significantly by 74.74% and 84.21% compared to ChC, respectively with disease severity of 14.33% and 6.22. The most pronounced decrease in 89.47% of infection compared to ChC with a disease severity of 2.88% was detected in GB+CHT-treated plants.

### 2.2. Plant Growth

**Figure 2:**
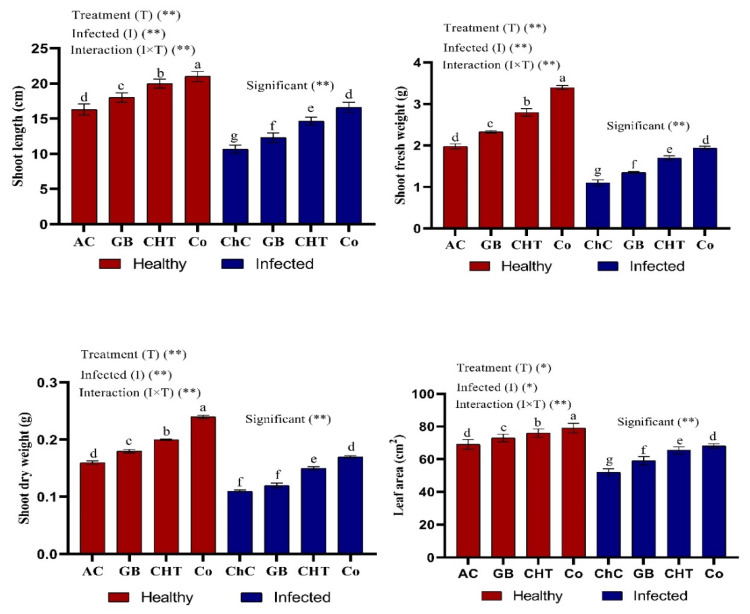
Effect of foliar spray by glycine betaine (GB), chitosan (CHT) and combination (Co) on plant growth of cucumber plant under absolute control (AC) and CMV infection (challenge control, ChC) after 21 days of inoculation. Means with the different letters (a, b, c, d, e, f, g) are significantly different at the 0.05 level among treatments according to Tukey’s test. Vertical bars represent means of ten independent determinations ± standard error (SE). * and ** indicate significant and highly significant difference according to two-way ANOVA with Tukey’s post hoc test.

Figure 2 shows that CMV-infected cucumber plants showed significant decreases in shoot length, fresh and dry weight of shoots and leaf area as compared with absolute control plants. On the other hand, treatment of infected plants with GB, CHT and GB+CHT resulted in significant increases in all of the above-mentioned morphologic characteristics compared to challenge control (infected plants). Moreover, all treatments showed a significant increase in morphologic characteristics compared to absolute control plants. Specifically, combined treatment (GB+CHT) resulted in the highest increase in healthy and infected plants followed by CHT alone and GB alone.

### 2.3. Photosynthetic Pigments and MDA Content

**Figure 3:**
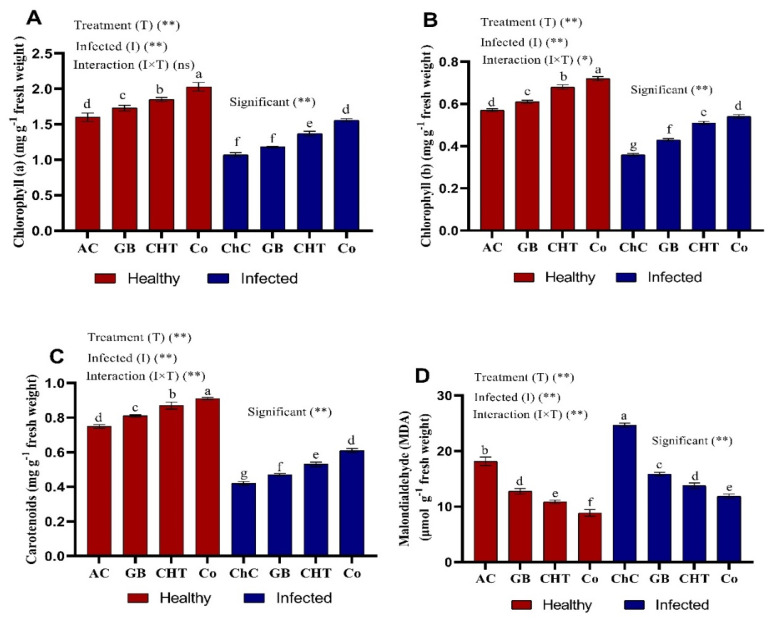
Effect of foliar spray by glycine betaine (GB), chitosan (CHT) and combination (Co) on photosynthetic pigments (**A**–**C**) and malondialdehyde (MDA) (**D**) content in leaves of cucumber plant under absolute control (AC) and CMV infection (challenge control, ChC) after 21 days of inoculation. Means with the different letters (a, b, c, d, e, f, g) are significantly different at the 0.05 level among treatments according to Tukey’s test. Vertical bars represent means of three independent determinations ± standard error (SE). *, ** and ns indicate significant, highly significant and ns, statistically insignificant difference according to two-way ANOVA with Tukey’s post hoc test.

Chlorophyll a, b and carotenoids content in leaves of cucumber plants show significant decreases about 33%, 37% and 44%, respectively after challenged with CMV compared to absolute control plants (Figure 3A–C). In addition, Chl a, Chl b and carotenoid content showed the highest values of about 46%, 50% and 45%, respectively, after treatment with a combination of GB+CHT compared to challenge control plants followed by CHT and GB. In addition, the content of Chl a, Chl b and carotenoid was significantly increased in the leaves of cucumber plants after treatment with GB, CHT and combination (GB+CHT) compared to stressed and non-stressed plants. Malondialdehyde (MDA) was significantly increased (about 36%) in leaves of cucumber challenged with CMV compared to absolute control plants. In addition, foliar spray with GB, CHT and GB+CHT showed reduction in MDA content about 36%, 44% and 52%, respectively in leaves as compared with challenged control plants (Figure 3D). The most pronounced reduction in MDA content was detected in leaves sprayed with GB+CHT (52%).

### 2.4. Endogenous Phytohormones

**Figure 4:**
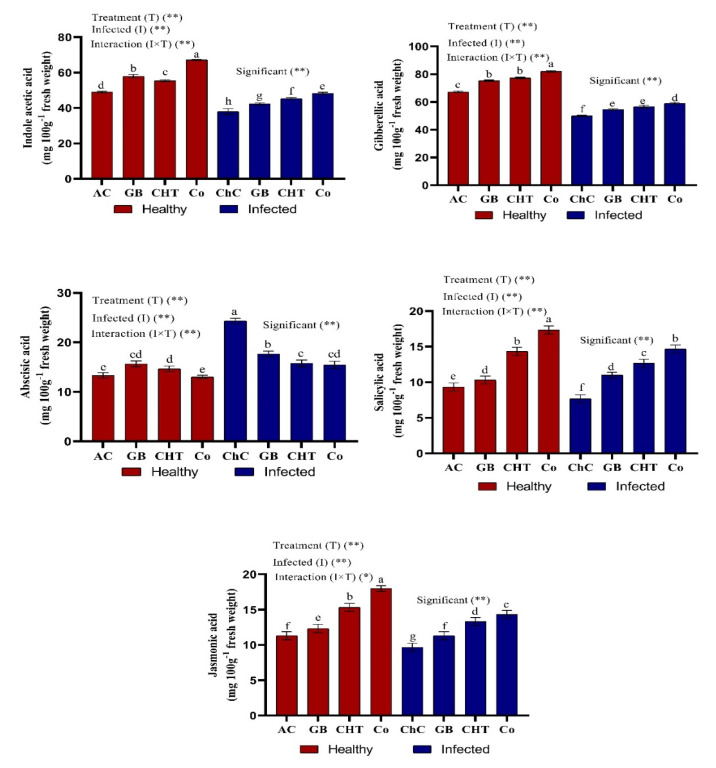
Effect of foliar spray by glycine betaine (GB), chitosan (CHT) and combination (Co) on phytohormones content in leaves of cucumber plant under absolute control (AC) and CMV infection (challenge control, ChC) after 21 days of inoculation. Means with the different letters (a, b, c, d, e, f, g) are significantly different at the 0.05 level among treatments according to Tukey’s test. Vertical bars represent means of three independent determinations ± standard error (SE). * and ** indicate significant and highly significant difference according to two-way ANOVA with Tukey’s post hoc test.

Cucumber plants infected with CMV showed significant reductions in the content of endogenous phytohormones such as indole acetic acid (IAA), gibberellic acid (GA_3_), jasmonic acid (JA) and salicylic acid (SA), but showed an accumulation of abscisic acid content (ABA) compared to absolute control plants (Figure 4). On the other hand, the treatment of infected cucumber leaves with combination of GB+CHT showed a maximum accumulation of IAA, GA_3_, JA and SA content and a minimum ABA content compared to challenge control plants followed by treatment with CHT and GB (Figure 4).

### 2.5. Osmolytes and Non-Enzymatic Antioxidants

**Figure 5:**
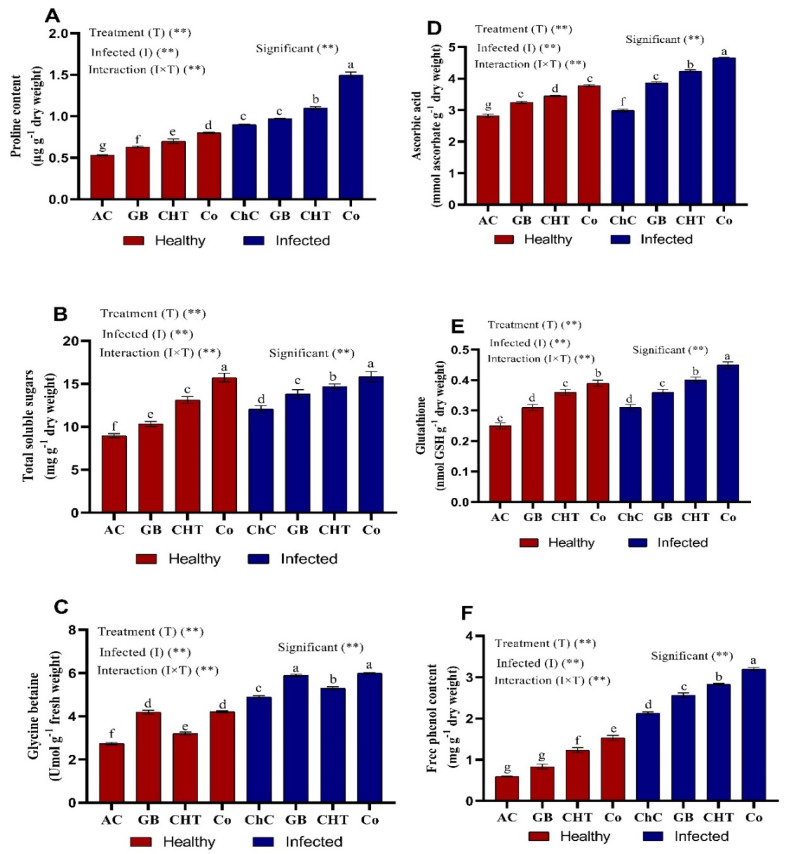
Effect of foliar spray by glycine betaine (GB), chitosan (CHT) and combination (Co) on osmolytes (**A**–**C**) and non-enzymatic antioxidants (**D**–**F**) content in leaves of cucumber plant under absolute control (AC) and CMV infection (challenge control, ChC) after 21 days of inoculation. Means with the different letters (a, b, c, d, e, f, g) are significantly different at the 0.05 level among treatments according to Tukey’s test. Vertical bars represent means of three independent determinations ± standard error (SE). ** indicate highly significant difference according to two-way ANOVA with Tukey’s post hoc test.

Healthy cucumber plants (non-CMV) treated with GB, CHT and a combination of GB+CHT showed a marginal increases in osmolytes (proline, soluble sugars and glycine betaine) (Figure 5A–C) and non-enzymatic antioxidants content (ascorbic acid, glutathione and phenol) compared to the absolute control plants (Figure 5D–F). On the other hand, osmolytes and non-enzymatic antioxidants were significantly increased in challenge plants (CMV-infected) treated with a combination of GB+CHT followed by CHT and GB compared with challenge control plants. Since the most pronounced increases in proline (67%), soluble sugars (31%), glycine betaine (22.5%), ascorbic acid (56%), glutathione (45%) and phenol (50%) were detected in infected plants treated with combination of GB+CHT compared to challenge control plants.

### 2.6. Antioxidant Enzymes

**Figure 6:**
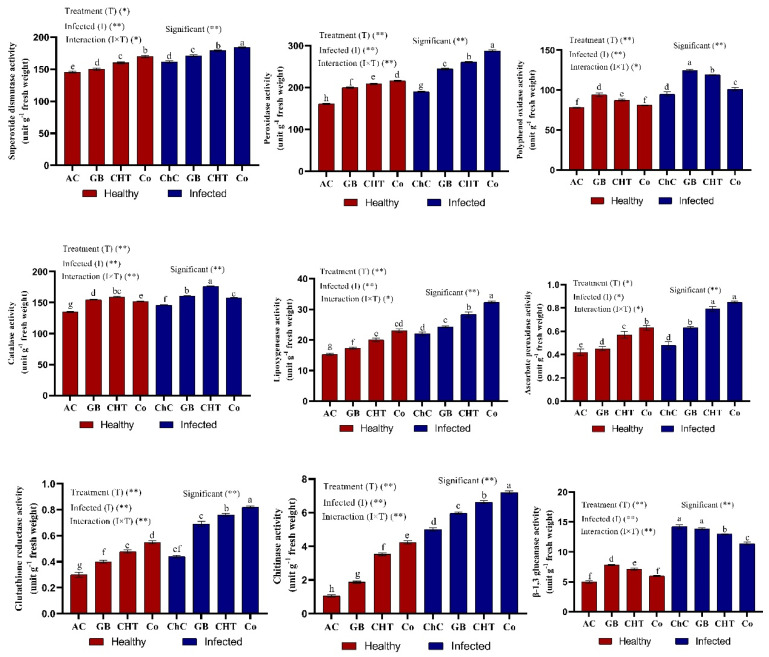
Effect of foliar spray by glycine betaine (GB), chitosan (CHT) and combination (Co) on enzymatic antioxidants content in leaves of cucumber plant under absolute control (AC) and CMV infection (challenge control, ChC) after 21 days of inoculation. Means with the different letters (a, b, c, d, e, f, g) are significantly different at the 0.05 level among treatments according to Tukey’s test. Vertical bars represent means of three independent determinations ± standard error (SE). * and ** indicate significant and highly significant difference according to two-way ANOVA with Tukey’s post hoc test.

The influence of viral infection and treatment with GB, CHT and GB+CHT on the activity of antioxidant enzymes (SOD, POX, PPO, CAT, LOX, APX, GR, chitinase and β-1, 3 glucanase) are shown in Figure 6. In the leaves challenged by CMV (challenge control plants), the activity of antioxidant enzymes activities was significantly increased compared to absolute control plants. In addition, the Tukey’s test statistical analysis showed that the activity of antioxidant enzymes (SOD, POX, PPO, CAT, LOX, APX, GR and chitinase) increased significantly in response to the treatment with combination of GB+CHT compared to challenge control plants. On the other hand, a significant decrease in the activity of β-1, 3 glucanase resulted from the treatment with a combination of GB+CHT compared to challenge control plants (Figure 6).

### 2.7. Gene Expression

**Figure 7:**
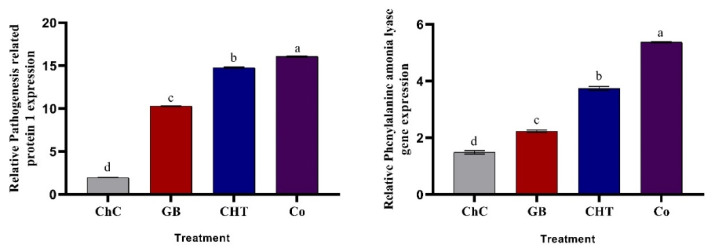
Effect of foliar spray by glycine betaine (GB), chitosan (CHT) and combination (Co) on the gene expression of relative pathogenesis related protein 1 (*PR1*) and relative phenylalanine ammonia lyase (*PAL1*) in leaves of cucumber plant under CMV infection and challenge control (ChC; untreated CMV-infected plants) after 4 days of inoculation. Means with the different letters (a, b, c, d) are significantly different at the 0.05 level among treatments according to Tukey’s test. Vertical bars represent means of three independent determinations ± standard error (SE).

The expression of pathogenesis-related genes, relative pathogenesis related protein 1 (*PR1*) and relative phenylalanine ammonia lyase (*PAL1*) exhibited a higher level of expression in cucumber plants treated with GB, CHT and combination after four days of CMV inoculation relative to the housekeeping gene (*ef1-∝*) (Figure 7). The most pronounced increases in *PR1* and *PAL1* expression were detected in plants treated with a combination of GB+CHT.

### 2.8. Correlation

**Figure 8:**
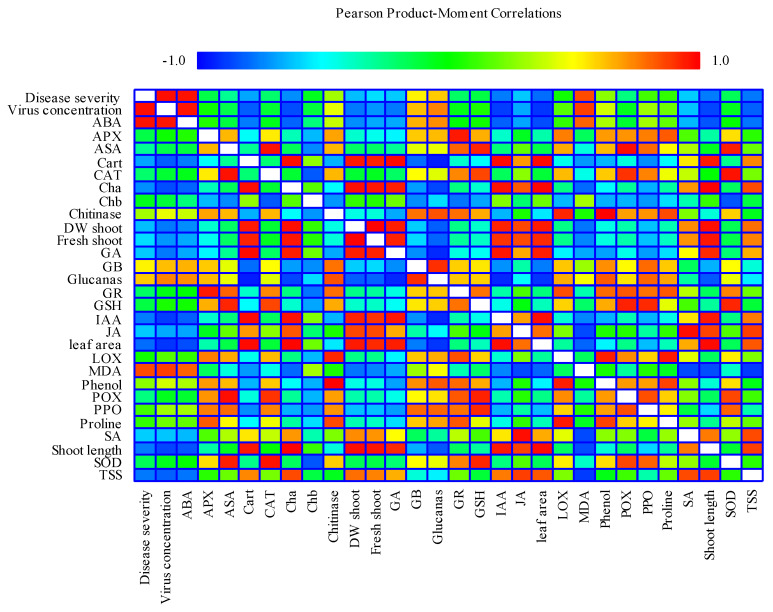
Heat map generated by Statgraphics XVII software Version 17.20 shows the relationship between quantitative statistical parameters depending on the mean values of various parameters, obtained in this study. Color scale displays the intensity of normalized mean values of different parameters. ABA: abscisic acid; APX: ascorbate peroxidase; ASA: ascorbic acid; Cart: carotenoids; CAT: catalase; Chla: chlorophyll-a; Chlb: chlorophyll-b; GA: gibberellic acid; GB: glycine betaine; GR: glutathione reductase; GSH: glutathione; IAA: indole acetic acid; JA: jasmonic acid; LOX: lipoxygenase; MDA: malondialdehyde; POX: peroxidase; PPO: polyphenol oxidase; SA: salicylic acid; SOD: superoxide dismutase; TSS: total soluble sugars; DW: dry weight; FW: fresh weight.

The result of the correlation analysis under CMV showed that disease severity, virus concentration and shoot length, FW shoot, DW shoot, leaf area, total photosynthetic pigments (Chl a, Chl b, Cart), TSS, LOX, chitinase, glucanase, proline, phenol, MDA, GB, GSH, IAA, GA, ABA, JA, SA, SOD, POX, APX, CAT, PPO had significant correlation (Figure 8). There were positive and significant correlations among disease severity, virus concentration and MDA, ABA. In contrast, there were negative and significant correlation between disease severity, virus concentration and shoot length, FW shoot, DW shoot, leaf area, total photosynthetic pigments (Chl a, Chl b, Cart), TSS, LOX, chitinase, glucanase, proline, phenol, GB, GSH, IAA, GA, JA, SA, SOD, POX, APX, CAT, PPO.

### 2.9. Multivariate Statistical Approach

**Figure 9:**
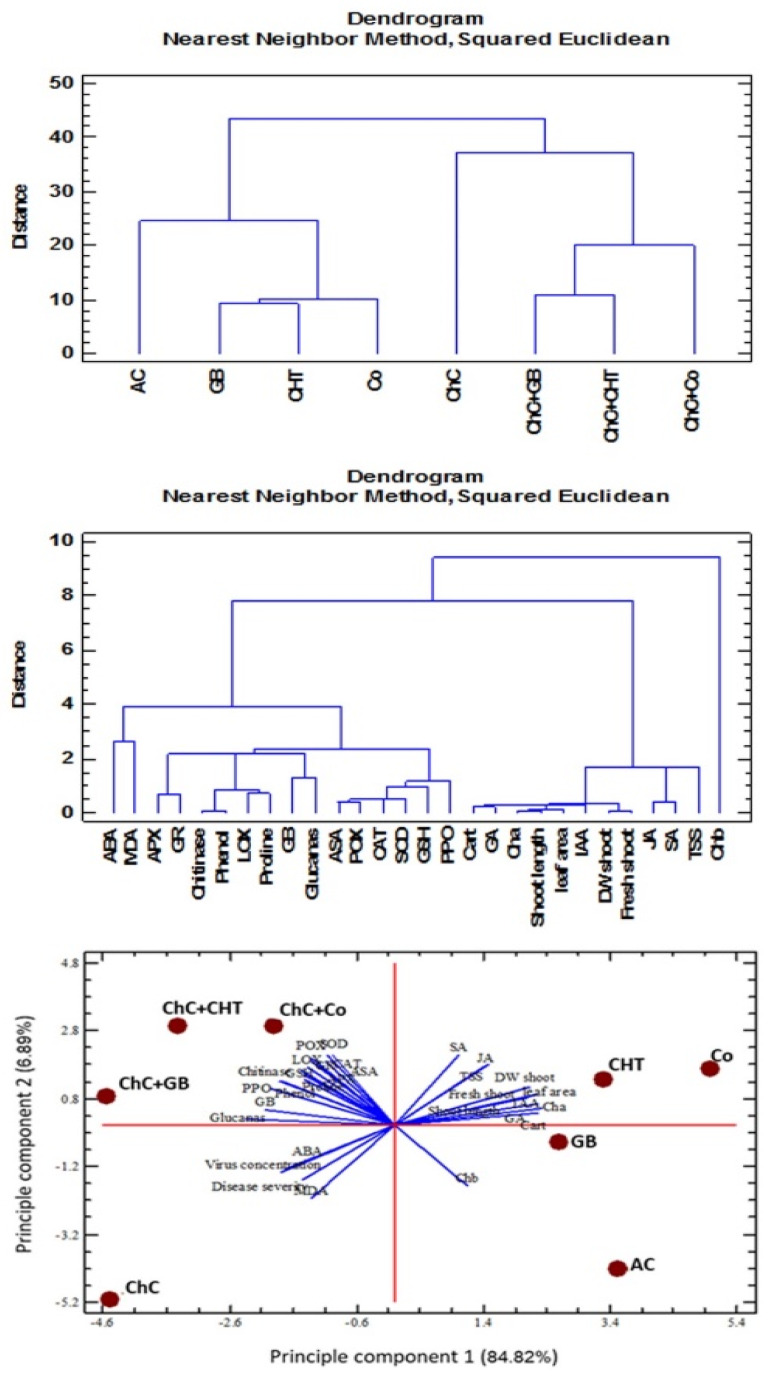
Hierarchical clustering (HCA) and principle component analysis (PCA) loading plot for glycine betaine (GB), chitosan (CHT) and combination (Co) treatment application under control and virus infection (ChC) generated by Statgraphics XVII software Version 17.20. ABA: abscisic acid; APX: ascorbate peroxidase; ASA: ascorbic acid; Cart: carotenoids; CAT: catalase; GA_3_: gibberellic acid; GB: glycine betaine; GR: glutathione reductase; GSH: glutathione; IAA: indole acetic acid; JA: jasmonic acid; LOX: lipoxygenase; MDA: malondialdehyde; POX: peroxidase; PPO: polyphenol oxidase; SA: salicylic acid; SOD: superoxide dismutase; TSS: total soluble sugars.

Hierarchical cluster analysis (HCA) was applied to the different traits from non-infected and infected plants with CMV by using Nearest Neighbor methods and Squared Euclidean distance as a measure of similarity (Figure 9). Mainly, two groups were formed by cluster analysis. Cluster 1 included (Chl b). However, cluster 2 converted to two sub group the first sub group (total photosynthetic pigments (Chl a, Cart), GA, shoot length, FW shoot, DW shoot, leaf area, IAA, JA, SA, TSS), while the second sub group (LOX, chitinase, glucanase, proline, phenol, MDA, GB, GSH, ABA, SOD, POX, APX, CAT, PPO).

HCA was also applied to different treatments on the basis of physiochemical parameters and non-stressed, stressed lead conditions (Figure 9). From the HCA results, it was found that mainly two clusters are formed: cluster 1 (non-infected) which are further sub-grouped into various clusters. AC, GB, CHT and Co are included in the same sub-group and cluster 2 (infected plant with CMV), ChC, ChC–GB, ChC–CHT and ChC–Co are included in the same cluster which can be attributed to the less variations of physiochemical. PCA was also conducted to different treatment on the basis of physiochemical parameters and non-infected, infected plant (Figure 9). The principle components were able to explain 96.32% (84.82% and 6.89%) of the total variance.

## 3. Discussion

In general, plants can tolerate the diseases infection through a wide range of cellular processes; (i) up- or down-regulation of different genes; (ii) changes in pathway levels of various compounds, like reactive oxygen species (ROS); (iii) stimulation of various transcription factors activity, regulation of defense genes and pathogenesis related proteins, (iv) stimulation of protective signaling enzymes and phytohormones such as SA, JA, auxins, cytokinins, abscisic acid and gibberellic acid [40,41]. The present findings show that GB and CHT can protect cucumber plants against CMV not only by the reduction of symptom and disease severity, but also by decreasing viral titer [42].

The maximum inhibition in infection and disease severity was detected in plants treated with combination of GB+CHT, followed by CHT and GB (Table 1). Thus, the inhibition of infectivity could be attributed to the potential of GB and CHT to prevent CMV replications by dissolving or penetrating the virus coat and denaturation of their proteins and nucleic acids [28]. The antiviral effect of GB and CHT appears to involve direct inhibition of virus replication or indirectly through inducing systemic resistance of the host plant against the virus [27]. One consequence of application of GB and CHT is an increase in plant growth under control (Figure 2), even in plants under stress, indirectly because of their high anti-pathogenic activity and directly through the (i) stimulation phytohormones biosynthesis, (ii) stimulation of absorption and solubilization of soil nutrients, (iii) stimulation the hardness of roots and their growth, (iv) improvement the carbohydrate metabolism levels and photosynthesis [28,42].

CHT was shown to induce the synthesis of callose in plant cells and the concentration of callose in extracts from treated leaves was found to correlate with the resistance to infection with PVX [28]. The role of callose in limiting of viral infection suggested that deposits of callose in pores of the phloem sieves appearing during plant defense responses serve as mechanical barriers preventing viral particles from traveling along vessels [43]. It was also suggested that extracellular callose deposited around plasmodesmata in the form of the so-called callose collar constrains the cell-to-cell transport of viruses [43].

In cucumber plants (Figure 3A–C), infection with the virus has caused a marked decrease in chlorophyll a, b and carotenoids content compared to healthy control plants. Similar results found that CMV infection caused reduction in photosynthetic pigments and plant growth in tomato [44] and cucumber [7,45] plants. The reduction in chlorophyll content in virus-infected plants can be caused by the stimulation of specific cellular enzymes such as chlorophyllase [46] or by the effect of the virus on pigment synthesis [47]. The cucumber plants treated with GB, CHT and the combination of GB+CHT showed an improvement in chlorophyll content, indicating that the GB and CHT can destroy the virus, thereby enhancing the host’s resistance to disease. On the other hand, it was found that the chlorophyll content of cucumber leaves treated with GB and CHT treatment is lower than in healthy control, indicating that GB and CHT may not be able to completely suppress the virus’ chloroplast damage [48].

Increased MDA content was detected in CMV-infected cucumber plants compared to control plants. An increase in MDA content was observed in CMV-infected cucumber plants as opposed to control plants. The oxidative stress induced by CMV and zucchini yellow mosaic virus (ZYMV) infected plants (*Cucumis sativus* and *Cucurbita pepo*) undergo enhanced peroxidation of polyunsaturated fatty acids, leading to an advanced disintegration of biomembranes [49]. Treatment with GB, CHT and combination decreased MDA content in infected plants compared to challenge control plants. CHT has a positive ionic charge which allows it to easily bind to negatively charged lipids, metal ions, proteins and macromolecules. Although it has positive ionic charges, chitosan, via changes in chromatin, stimulates defense genes and helps to reduce lipid peroxidation [50]. MDA decreased by GB treatment, but LOX increased [51]. Previous studies have shown that both LOXs in plants play a significant role in catabolism of phospholipids by inducing a lipolytic cascade in membrane degradation during stress [52].

Treatment of infected cucumber leaves with combination of GB+CHT showed a maximum accumulation of IAA, GA_3_, JA and SA content and a minimum ABA content compared to challenge control plants followed by treatment with CHT and GB (Figure 4). GB, salicylic acid (SA) and JA are important natural hormones that have a plant resistance function against infectious viruses. Several studies have found that SA inhibits the replication of the tomato bushy stunt virus (TBSV) through competitive glyceraldehyde 3-phosphate dehydrogenase (GAPDH) bindings in one cell. SA accumulations can lead to its direct GAPDH binding, effectively preventing RNA virus interaction paving the way for TBSV replication suppression [53]. SA also prevents the movement and replication of viruses through another mechanism that involves the intervention of the alternative pathway to mitochondria oxidase (AOX). In particular, AOX acts as an inhibitor to combat the TMV, PVX and CMV resistance caused by SA [54]. In addition, the virus infection caused a reduction in phytohormones compared to absolute control plants. Auxin is a crucial phytohormone, which is directly disrupted by viral components [55]. Disruption of the pathway to GA_3_ biosynthesis is also closely related to symptoms of viral infections. GA_3_ participates in the division and elongation of the cells [56]. In addition, the JA biosynthesis gene enhanced resistance and reduced local resistance to TMV [57]. In addition, GB and CHT increased accumulation of JA which correlated with the increase in LOX enzyme activity in infected cucumber plants. The LOX pathway leads to the mechanism of plant defense against biotic stress by synthesizing various compounds with signaling functions such as JA [58].

Accumulation of ABA content in CMV-infected cucumber plants as opposed to control plants. Similar findings showed that the increase in ABA content in CMV-infected *Nicotiana benthamiana* [59]. Virus-infected plants, ABA enters the antiviral silencing pathway interfering with virus accumulation and the micro RNA (miRNA) pathway through which ABA affects miRNAs ‘maturation and stability [60]. ABA also induces the deposition of calluses in plasmodesmata, a mechanism which restricts the movement of viral cells into cells [61]. Virus movement from cell to cell occurs through the vascular phloem through intercellular plasmodesmata connections. ABA impedes virus movement by inhibiting β-1,3-glucanase, which degrades callose [62].

In this study the GB and CHT caused accumulation in SA content, expression of *PR1* and *PAL1* genes (Figure 7) and phenolic content (Figure 5) in challenged plants with CMV. The level of SA production depends on how the *PAL* gene is expressed [63]. PAL is a key regulator of the phenylpropanoid pathway that participates in the production of phenolic compounds with a significant range of biologic functions [64]. SA has the ability to induce acquired resistance in many plants against various necrotizing and systemic viral, fungal and bacteria and increased the expression of *PR* genes [65]. SA signaling is activated to suppress viral, bacterial and fungal pathogens invasion in many plant species through up—regulation of pathogenesis—related protein (*PR*) genes expression and the development of SAR [66].

The ascorbic acid, glutathione and phenolic content increased in the inoculated (viral control as challenge control) plants compared to the corresponding levels of the healthy control plants (absolute control). In addition, treating cucumber plants with GB, CHT and combination increased all the above content above that of healthy and virus-infected plants (Figure 5D–F). Similar findings showed that infected plants with pepper mild mottle virus preserved their total glutathione content during the infection at values close to controls [67]. Modulation of the cellular glutathione content to transmit information via various signaling mechanisms has been described [68]; glutathione was involved in the protein protection mechanism against oxidative damage and defense-related gene regulation [69]. The increment of cellular GSH can significantly suppress the symptoms of viral disease, and in some cases, also the multiplication of viruses [68].

Upon infection by viruses and treatment with GB, CHT and combination, the higher concentration of phenolic compounds in the host plants could contribute to enhancing the strength of the host cell walls by the synthesis of lignin and suberin known to be involved in the formation of physical barriers against the spread of pathogens [70]. In addition, phenolic, such as SA are antifungal at high concentrations and helps to defend the host plants against fungal invasion [65].

CMV infection (challenge control) increased the activities of antioxidant enzymes (SOD, POX, PPO, LOX, APX, GR, chitinase and β-1, 3 glucanase) compared to those of healthy plants (absolute control). In contrast, the treatments with GB, CHT and combination increased the enzyme activities more than in virus-infected plants (Figure 6). As stated by Vitti, Pellegrini, Nali, Lovelli, Sofo, Valerio, Scopa and Nuzzaci [45], CMV infection contributes to ROS, which can react with proteins, lipids and deoxyribonucleic acid that cause oxidative damage and impair plant cell normal functioning. In order to overcome these effects, plants evolve antioxidant defense systems that include both enzymatic and non-enzymatic components (i) decrease ROS generation and accumulation and (ii) decrease oxidative damage during infection [45].

POD, PPO and PAL defense enzymes are generally considered essential in fostering host resistance because they can catalyze the final step of lignin biosynthesis [71] and can oxidize phenolic compounds to quinones [72], and these quinones are virus toxic [73]. In addition, the increase in the resistance of plants treated with GB and CHT may be due to the expression of genes encoding the pathogenesis-related (PR) proteins such as hydrolytic enzymes chitinase and β- 1,3-glucanase which can destroy fungal and bacterial cell walls [65], increased the expression of phenylpropanoid enzyme encoding genes, such as phenylalanine ammonia lyase (PAL), which contribute to the formation of phytoalexins and other phenolic compounds; enhanced peroxidase activity which is essential for lignification and capable of interlinking the cell wall protein [74]. CAT scavenges unstable ROS into less harmful and more stable components like O_2_ and water [75]. Under virus infection and treated plants, CAT activity was increased and interact with the virus, especially certain virus elements, such as CMV 2b protein [76]. This promotes the persistence of H_2_O_2_ in the cell, which may in effect induce SA accumulation [77], as evidenced by the substantial increase in SA levels. LOX also increased after CMV infection and treatment with GB and CHT. Similar results reported by Lavanya and Amruthesh [42] found that exogenous application of GB against *Sclerospora graminicola* in Pearl Millet inducing LOX expressions for protecting pathogen which could lead to desaturation of lipid peroxidation products by increasing membrane stability and genes involved in stress defense mechanism via the octadecanoid pathway.

## 4. Materials and Methods

### 4.1. Plant Materials and Chemicals

Seeds of cucumber plants (*Cucumis sativus* L. cv. Beta–alpha) were obtained from Agricultural Research Center, Ministry of Agriculture, Giza, Egypt. The glycine betaine (GB) solutions were prepared using distilled water and GB granular powder (≥98% perchloric acid titration, MW 117.15, Sigma-Aldrich). Chitosan (CHT (50–190 kDa) was obtained from Sigma-Aldrich. CHT (1 g) was dissolved under constant stirring overnight in purified water (40 mL) containing 1 M acetic acid (9 mL). The pH was adjusted to 5.4 and then the solution was complete to 100 mL of distilled water (concentration 1%). Two drops of Tween 80 were applied to each solution before spraying to increase distribution on the leaves.

### 4.2. CMV Inoculation

The CMV isolate (Gera-EG, Accession no. JQ013954) [36] used in the study was maintained on *Nicotiana glutinosa*. The virus was checked on *Chenopodium amaranticolor* L. The inoculum of the virus was prepared using sterilized pestle and mortar by grinding *Nicotiana glutinosa* fresh leaves in phosphate buffer (100 mM, pH 7.5). The pulp was pressed through two layers of cheesecloth and the filtrate was centrifuged for 10 min at 10,000× *g*. Cotyledon leaves of cucumber plants were dusted lightly with carborundum (600 mesh). The supernatant containing the virus was used after dilution 10^–1^ with the same buffer as a virus inoculum.

### 4.3. Experimental Design

Cucumber plants were grown in 30 cm diameter pots containing a sterile soil mixture of clay: sand: peat moss with ratio of 1:1:1 (w:w:w) and left to grow for 3 weeks under greenhouse conditions (12/12 h day/night photoperiod at 25 °C). After that, the plants were divided into eight groups. Each group consisted of three replicates in which the replicate consisted of 10 plants per group. The first group was sprayed with water considered as a control without any treatment (absolute control). Second, third and fourth groups were treated by spraying the leaves (about 10 mL plant^−1^) with GB (50 mM), CHT (1%) and combination between GB+CHT (1:1 *v/v*), respectively. The fifth group was inoculated with CMV as a positive control (challenge control) at the same time that the other groups were inoculated. In the sixth, seventh and eighth groups, the plants were sprayed with GB, CHT and GB+CHT, respectively, and after 3 days of spraying, the virus was inoculated. All leaves were mechanically inoculated. The inoculum was prepared from contaminated cucumber leaves, ground in a mortar containing phosphate buffer (0.1 M, pH 7.0) (1:2 *w/v*); the homogenate was filtrated through two layers of muslin, and the leaves of healthy plants were dusted with carborundum and gently rubbed with a cotton swab that had previously been dipped into virus inoculums suspension. The percentage of infected plants and the severity of symptoms were assessed three weeks after inoculation using the following rating scale: 0 = no symptoms, 2 = Vein clearing, 4 = Mild mosaic, 6 = severe mosaic, 8 = severe mosaic and blisters 10 = severe mosaic, blisters and malformation [78]. Disease severity values were calculated using the following formula according to Yang, et al. [79]:

(1) $$\mathrm{DS}\;\left(\%\right)=\frac{\sum \left(\mathrm{Disease}\;\mathrm{grade}\times \mathrm{Number}\;\mathrm{of}\;\mathrm{plants}\;\mathrm{in}\;\mathrm{each}\;\mathrm{grade}\right)}{\left(\mathrm{Total}\;\mathrm{number}\;\mathrm{of}\;\mathrm{plants}\times \mathrm{Highest}\;\mathrm{disease}\;\mathrm{grade}\right)}\times 100$$

### 4.4. Enzyme-Linked Immunosorbent Assay (ELISA)

Indirect ELISA was used to estimate the accumulation of CMV according to the method defined by Zehnder, et al. [80] with some modifications by Derbalah and Elsharkawy [81]. For the ELISA test, the third leaf from the top of the cucumber plant was chosen. After 21 days of inoculation, leaf samples were collected, grounded in a 50 mM carbonate buffer (pH 9.6) and applied to microtiter plates at a final dilution of 1 g tissue: 10 mL buffer. The plates were held at 4 °C overnight and then washed three times with phosphate-buffered saline containing Tween (PBS-T). Anti-CMV (Loewe Biochemica GmbH, Germany) was applied to the plates, dissolved in PBS at a concentration of 1 g mL^−1^. The plates were then incubated at 37 °C for 1.5 h and washed with PBS. Goat anti-rabbit immunoglobulin conjugated with alkaline phosphatase was diluted (1:7500) and added to the plates. The plates were incubated for 1 h at 37 °C and then washed three times with PBS-T. The substrate (*p*-nitrophenyl phosphate at 1 mg mL^−1^ in 10% diethanolamine, pH 9.8) was then applied and the production of the reaction at room temperature was permitted. The absorbance was measured at a wavelength of 405 nm using a Sunrise™ TECAN, Switzerland microplate reader. The ELISA values were indicated to be positive for more than twice of healthy plant values. ELISA experiment was conducted three times and three replicates (two leaves for each replicate) were used for each test.

### 4.5. qRT-PCR Analysis

After 4 days of inoculation, leaf tissues of CMV inoculated cucumber plants treated with GB, CHT and GB+CHT and untreated control plants were sampled. Wang, et al. [82] and Derbalah and Elsharkawy [81] defined a protocol used to conduct the quantitative (q) RT-PCR reaction. qRT-PCR was carried out using gene specific primers [83] and the normalization of the target gene quantity was obtained by standardizing the abundance of the constitutive *ef1-∝* gene (alpha elongation factor) as shown in Table 2. The RT-PCR technique was carried out using the 7000 RT-PCR method and the data collected were analyzed using the ABI PRISM 7000 (Applied Biosystems) program. This software calculates the normal expression rate (standard curve approach for estimating relative genes) and p values to estimate related statistical changes. The comparative 2-CT technique was conducted as Livak and Schmittgen [84]. It was used to calculate relative gene expression according to the values of the cycle threshold (CT) obtained from the program ABI PRISM 7000. To get correct data this experiment was repeated three times.

### 4.6. Measurements

#### 4.6.1. Growth Indices

Three weeks after inoculation, 10 seedlings were randomly selected from each treatment to determine shoot length, fresh and dry weight of shoot and leaf area. Shoots were dried in oven at 80 °C for at least 72 h until constant weight and was recorded. For the study of biochemical changes, the youngest entirely formed leaves were harvested from both control and treated plants.

#### 4.6.2. Biochemical Measurements

##### Estimation of Photosynthetic Pigments

Samples of 0.5 g fresh leaves were ground with pestle mortar in liquid nitrogen. The samples were mixed with acetone (80%) and the mixture was centrifuged at 20 °C for 5 min at 10,000× *g*. In order to estimate chlorophyll-a, chlorophyll-b [85] and carotenoids [86], the filtrate was measured at wavelengths of 470, 652 and 665 nm using a spectrophotometer.

The contents of chlorophyll a, b and carotenoids in the plant tissue were calculated using the following equations:

mg chlorophyll a g^−1^ tissue = 11.63 (λ 665) − 2.39 (λ649) (2)

mg chlorophyll b g^−1^ tissue = 20.11(λ649) − 5.18 (λ665) (3)

mg carotenoid g^−1^ tissue = 1000 × (λ 470 − 1.82 Chl a − 85.02 Chl b/198) (4)

### 4.7. Determination of Lipid Peroxidation

The lipid peroxidation products as described by Heath and Packer [87] were measured and quantified in terms of malondialdehyde (MDA). In 2 mL of 0.1% (*w/v*) trichloroacetic acid (TCA), 0.5 g of the cucumber leaves were homogenized, followed by 20 min of centrifugation at 12,000× *g*. The collected supernatant (1 mL) was mixed with an equivalent TCA volume (10%) containing 0.5% (*w/v*) TBARS, heated at 95 °C for 30 min, and then cooled in ice and then the absorption of supernatants was measured at 532 and 600 nm. After subtraction of the non-specific absorbance (600 nm), the concentration of MDA was calculated by its molar extinction coefficient of 155 mM^−1^ cm^−1^.

### 4.8. Extraction, Separation and Estimation of Hormonal Content

#### 4.8.1. Extraction

Endogenous hormones (IAA, GA_3_, ABA, JA, SA) were extracted from the terminal buds of plants and determined by Knegt and Bruinsma [88] method as follows:

Five g of fresh leaves was collected and put overnight at 4 °C in dark in 80% redistilled cold MeOH (10 mL MeOH per g FW). Filtering of the extract by filter study (Whatman No.1) and then washed with 5 mL of 80% MeOH. The extract was then evaporated by rotary evaporator into aqueous phase. The residue, i.e., aqueous phase, was dissolved by 5 mL phosphate buffer (0.1 M, pH 8) and held at −18 °C for 24 h. The defrosted extract was centrifuged at 4 °C at a rate of 12,000× *g*, polyvinylpyrrolidone (PVP) was applied and filtered through Whatman No.1, then the filtrate was washed by 7 mL phosphate buffer (0.1 M, pH 8) and the total volume complete up to 30 mL. Partition extract was carried out against diethyl ether (1:2 volume) two times. Organic phase was discarded each time. Partition extract was performed two times against diethyl ether (1:2). Through time, organic phase was discarded. The aqueous phase pH was changed to pH 2.5 using 5-N HCl and partitioned twice with diethyl ether and discarded the aqueous phase. The organic phase was evaporated to dry film and then dissolved with 5 mL H_2_O (0.1 N acetic acid) and pH with 1 N acetic acid was changed again to 2.5.

#### 4.8.2. Separation

The reversed phase C18 sep-pack cartridge was used to isolate the endogenous hormones of plants (IAA, GA_3_, ABA, JA, SA) as described by Lee, et al. [89].

#### 4.8.3. Estimation

IAA, GA_3_, ABA, JA, SA were estimated by HPLC as follows:

Water U6 K HOLC;

Column: Bondapak C_18_;

Dimension: 3.9 × 300 mm;

Mobil phase: MeOH super purity −2% acetic acid;

Flow rate: 1.0/min;

Detection: UV waters 486–254 nm.

### 4.9. Proline Content

The content of proline was analyzed using the colorimetric method of Bates, et al. [90]. In 10.0 mL of 3.0% (*v/v*) sulfosalicylic acid, 500 mg of dried cucumber leaves was homogenized. The mixture was then centrifuged at 10,000× *g* for 10 min. 2 mL of the supernatant was moved to a test tube and then added 2 mL of freshly prepared acid–ninhydrin solution. The test tubes were incubated in a water bath at 90 °C for 30 min. In each test tube, the reaction was finished in an ice-bath. Each reaction mixture was collected for 15 sec using 5 mL of toluene and vortex-mixed. At room temperature, the test tubes were allowed to stand in the dark for 20 min to isolate the toluene and aqueous phases. Upper toluene phase was then carefully collected into a cuvette and its absorption was read with a spectrophotometer at 520 nm.

### 4.10. Total Soluble Sugars Analysis

200 mg dried cucumber leaves were homogenized with 5 mL of 96% *v/v* ethanol, and then washed with 5 mL of 70% *v/v* ethanol. For 10 min, samples were centrifuged at 3500x *g*. Then, 0.1 mL of the extract was reacted by heating for 10 min in a water bath with 3 mL of freshly prepared anthrone reagent (150 mg anthrone + 100 mL of 72% (*v/v*) sulfuric acid). Cooling was completed and the mixture was absorbed at 652 nm using spectrophotometer as descried by Irigoyen, et al. [91] method.

### 4.11. Glycine Betaine Analysis

In 20 mL test tube, 0.5 g fresh cucumber leaves were ground in 5 mL of the toluene–water mixture (0.05% toluene). Each tube was mechanically shaken for 24 h at 25 °C. After filtration 0.5 mL of extract was mixed with 1 mL of 2-N HCl solution then applied and shaken in an ice-cold water bath for 90 min. 0.1 mL of potassium tri-iodide solution (containing 7.5 g iodine and 10 g potassium iodide in 100 mL of 1-N HCl), 2 mL of ice-cooled water was added and then 10 mL of 1, 2 dichloroethane (chilled at −10 °C) was poured into it. Two layers were separated by going through continuous airflow for 1–2 min, the upper aqueous layer was discarded, and the organic layer was read at 365 nm optical density according to the method of Grieve and Grattan [92].

### 4.12. Ascorbic Acid Analysis

500 g of dried cucumber leaves was extracted in 10 mL of 6% (*w/v*) trichloroacetic acid (TCA). The extract was then combined with 2 mL of 2% (*w/v*) dinitro-phenylhydrazine, followed by one drop of 10% (*w/v*) thiourea dissolved in 70% (*v/v*) ethanol. Then, the mixture was boiled in a water bath for 15 min. 5 mL of 80% (*v/v*) H_2_SO_4_ was applied at zero °C after cooling the samples at room temperature. Using spectrophotometer, the absorbance was measured at 530 nm to determine the content of ascorbic acid (ASA) as described by the method of Mukherjee and Choudhuri [93].

### 4.13. Glutathione (GSH) Content

50 mg of dried cucumber leaves were extracted in tube contain 2 mL of 2% (*v/v*) metaphosphoric acid and then centrifuged at 17,000× *g* for 10 min. The supernatant (0.9 mL) was mixed with 0.6 mL of 10% (*w/v*) sodium citrate. A mixture of 700 μL 0.3-mM NADPH, 100 μL 6-mM 5.5′-dithio-bis-2-nitrobenzoic acid, 100 μL distilled water and 100 μL extract was stabilized for 3–4 min at 25 °C. Then, GSH reductase (10 μL of 50 units mL^−1^) was then applied to the extract and absorbance at 412 nm was read using a spectrophotometer to measure the GSH content from the standard curve as defined by Griffith [94].

### 4.14. Free Phenol Analysis

Free phenols were analyzed using Folin–Ciocalteu reagent and sodium carbonate solution according to Galicia, et al. [95]. One hundred milligrams of dried cucumber leaves were mixed with 6.5 mL methanol (50%). The samples were vortexed, allowed to stand for 90 min in the dark at room temperature and centrifuged at 12.000× *g* for 5 min. One milliliter of the supernatant was added to 0.8 mL of 25% Folin–Ciocalteu reagent, 5 mL of 85% phosphoric acid and 10 mL of concentrated hydrochloric acid. The tubes incubated at 42 °C for 9 min. Then, the absorbance was read at 765 nm using spectrophotometer.

### 4.15. Enzymes Activity

#### Extraction and Analysis

Leaves of cucumber were used for estimation of superoxide dismutase (SOD), peroxidase (POX), polyphenol oxidase (PPO), catalase (CAT), lipoxygenase (LOX), ascorbate peroxidase (APX), glutathione reductase (GR) and chitinase and β -1,3 glucanase enzymes. In this regard, 2 g of fresh cucumber leaves were mixed with 10 mL of phosphate buffer (0.1 M, pH 6.8), then centrifuged in an ice-cooled centrifuge at 2 °C for 20 min at 15,000× *g*. The supernatant (containing the enzymes) was taken as enzyme source.

The activity of superoxide dismutase (SOD: EC 1.15.1.1) was calculated by measuring the inhibition of pyrogallol auto-oxidation using a method defined by Marklund and Marklund [96]. The solution (10 mL) was mixed with 3.6 mL distilled water, 0.1 mL enzyme, 5.5 mL phosphate buffer (0.1 M, pH 6.8) and 0.8 mL 3-mM pyrogallol (dissolved in 10-mM HCl). The reduction rate of pyrogallol was measured with UV- spectrophotometer at 325 nm (Model 6305, Jenway).

Peroxidase (EC 1.11.1.7) and polyphenol oxidase (EC1.14.18.1) were analyzed using solution containing 5.8 mL phosphate buffer (0.1 M, pH 6.8), 0.2 mL of the enzyme extract and 2 mL of 20-mM H_2_O_2_. After addition of 2 mL of 20-mM pyrogallol, the increase in the absorbance as pyrogallol was determined using UV-spectrophotometer (Model 6305, Jenway) within 60 s at 470 nm and 25 °C [97]. One unit of the enzyme activity was defined as the amount of the enzyme that catalyzed the conversion of one micromole of H_2_O_2_ per min at 25 °C. The blank sample was prepared using buffer instead of enzyme extract.

Lipoxygenase activity (LOX; EC 1.13.11.12) was assayed according to the method of Todd, et al. [98]. In 40 mL sodium phosphate buffer (0.1 M, pH 6.8), the normal assay mixture contained 200 μL of 20 to 40 μL of linoleic acid (Aldrich). Then, 0.2 mL of LOX extract was applied to 1 mL of normal assay mixture in a cuvette. One unit of LOX activity was identified as the amount of enzyme that caused an increase of 234 nm in absorption.

Catalase activity (CAT; EC 1.11.1.6) was analyzed using Chen, et al. [99] method. The reaction mixture with final volume of 10 mL containing 9.96 mL H_2_O_2_ phosphate buffer (0.1 M, pH 6.8) was added to the reaction mixture with final volume of 10 mL containing 40 μL enzyme extract. CAT activity was determined via the rate change of H_2_O_2_ absorbance in 60 s with a UV-spectrophotometer at 250 nm.

Ascorbate peroxidase activity (APX: EC 1.11.1.11) was assayed by the method of Nakano and Asada [100]. The reaction mixture contained potassium phosphate buffer (0.1 M, pH 6.8), 5 mM ascorbate, 0.5-mM H_2_O_2_ and enzyme extract. Addition of started the reaction. The rates were corrected for nonenzymatic oxidation of ascorbate by the inclusion of reaction mixture without enzyme extract. The absorbance read at 265 nm (e = 13.7 mM^–1^ cm^–1^).

Glutathione reductase (GR: EC 1.6.4.2) activity was assayed according to Jiang and Zhang [101] after oxidation of NADPH at 340 nm (extinction coefficient 6.2 mM cm^−1^) for 1 min.

Chitinase (EC 3.2.1.14) was carried out according to the method of Monreal and Reese [102] using colloidal chitin as the substrate and dinitro salicylic acid as reagent to measure reducing sugars. The determination optical density was determined at 540 nm.

β-1, 3 glucanase (E.C. 3.2.1.39) was determined according to the method of Abeles and Forrence [103]. Laminarin was used as the substrate and dinitro salicylic acid as reagent. The optical density was read at 500 nm.

### 4.16. Statistical Analysis

The experimental design was entirely randomized, and statistical analysis was performed using SPSS (Social Science version 26.00) statistical software at a significance level of 0.05 [104]. Quantitative analyses were obtained using one-way ANOVA, two-way ANOVA with Tukey’s post hoc test variance analysis with the parametric distribution of Levene’s study. The interval of confidence was set at 95% and the agreed error margin was set at 5%. Graphs were taken with GraphPad Prism 8. To show the relationship between quantitative statistical parameters, heatmap correlation, Hierarchical cluster and principal component analysis were estimated using Statgraphics XVII software Version 17.20.

## 5. Conclusions

**Figure 10:**
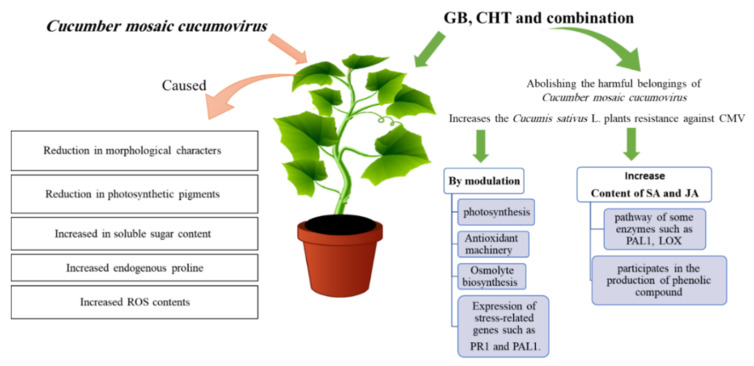
Model showing the role of glycine betaine (GB) and chitosan (CHT) in induce resistance in cucumber plant after CMV infection.

The goal of this study is to investigate the influence of viral infection on growth indices and metabolism of cucumber plants and also the effects of using GB, CHT and combination in the control of the infection with *Cucumber mosaic cucumovirus* on these plants. The study indicated that the application of GB and CHT, mainly when in combination, reduced disease severity and improved the plants resistance to CMV. This was evidenced by less severe symptoms, higher systemic resistance such as modulation of photosynthesis, antioxidant machinery, osmolyte biosynthesis and the expression of stress-related genes such as *PR1* and *PAL1*. The mechanism of GB and CHT effect is to enhance the plant defense against virus infection by increasing the content of SA and JA hormones which produced by the pathway of some enzymes such as PAL and LOX. PAL is a key regulator of the phenylpropanoid pathway that participates in the production of phenolic compounds which help in the plant defense (Figure 10). Therefore, the use of GB in combination with CHT is advised foe cucumber plants. Moreover, to our knowledge, this study is the first report to use GB to alleviate the adverse effects of virus infection.

## Figures and Tables

**Table 1:** Effect of foliar spray by glycine betaine (GB), chitosan (CHT) and combination (GB+CHT) on virus concentration, percentage of infection (%) and disease severity of cucumber plants leaves under CMV infection.

Treatments	Virus Concentration	Percentage of Infection (%)	Disease Severity (%)
Challenge control (ChC)	1.30 ± 0.04 a	95 ± 0.33 a	70 ± 0.24 a
GB + Virus	0.48 ± 0.01 b	24 ± 0.41 b	14.33 ± 0.38 b
CHT + Virus	0.35± 0.03 c	15 ± 0.25 c	6.22 ± 0.52 c
Combination (GB+CHT) + Virus	0.28 ± 0.02 d	10± 0.20 d	2.88± 0.33 d

Means (±SE standard error) followed by different letters (a, b, c, d) are significantly different from each other as indicated by Tukey’s test (*p* < 0.05).

**Table 2:** Forward and reverse primers sequence for *PR1, PAL1* and *ef1-∝* genes.

Gene	Forward Primer (´5–´3)	Reverse Primer (´5–´3)
*PR1*	TGCTCAACAA ATGCGAACC	TCATCCACCCACAACTGAAC
*PAL1*	ATGGAGGCAACTTCCAAGGA	CCATGGCAATCTCAGCACCT
*ef1-* *∝*	ACTGTGCTGTCCTCATTATTG	AGGGTGAAAGCAAGAAGAGC
